# Mental well-being in residents of monolingual and multilingual regions of Russia

**DOI:** 10.1192/j.eurpsy.2022.772

**Published:** 2022-09-01

**Authors:** R. Shilko, L. Shaigerova, O. Almazova, A. Dolgikh, O. Vakhantseva

**Affiliations:** Lomonosov Moscow State University, Psychology, Moscow, Russian Federation

**Keywords:** monolingual and multilingual regions, Russia, mental well-being, mental health

## Abstract

**Introduction:**

Despite numerous studies of the mental health and well-being of the population depending on various factors, insufficient attention is paid to the research of the relationship between well-being and mono- and multilingual regional specifics in multilingual and multicultural Russia.

**Objectives:**

This study aims to identify a possible relationship between mental well-being in some regions of the Russian Federation and monolingualism and multilingualism inherent in these regions.

**Methods:**

The study involved 966 participants (29.5% men and 70.5% women) aged 11 to 80 years (M=24.8; SD=12.19) from six regions of the Russian Federation: Crimea, Adygea, Bashkortostan, Sakha, Tatarstan and Kabardino-Balkarian region. The mental well-being of participants was assessed using the Warwick-Edinburgh Mental Well-being Scale (Tennant et al., 2006; Tennant et al., 2007).

**Results:**

The measures of mental well-being were compared among residents from the regions as following categories: native Russian language speakers in monolingual regions (144 participants); native Russian language speakers in multilingual regions (193 participants); native national language speakers in multilingual regions (325 participants); native Russian and national languages speakers in multilingual regions (304 participants). Using one-way analysis of variance (ANOVA), it was found that there were no significant differences in the assessments of mental well-being (F = 0.852; p = 0.521) among residents from the above categories.

**Conclusions:**

Residents who are native speakers in Russian and national languages and are living in Russia’s regions with monolinguism and multilinguism demonstrate no difference in mental well-being measures. The reported study was funded by the RFBR, project number 17-29-09167.

**Disclosure:**

No significant relationships.

